# Significance of C-reactive Protein Levels in Categorizing Upper and Lower Urinary Tract Infection in Adult Patients

**DOI:** 10.7759/cureus.26432

**Published:** 2022-06-29

**Authors:** Srikanth N Narayan Swamy, Raveendra K Jakanur, Shubha R Sangeetha

**Affiliations:** 1 Department of Internal Medicine, MS Ramaiah Medical College, Bangalore, IND; 2 Department of Pathology, Dr. B.R. Ambedkar Medical College, Bangalore, IND

**Keywords:** c-reactive protein, cystitis, bacterial infections, pyelonephritis, urinary tract infections

## Abstract

Background: Urinary tract infections (UTIs) are one of the most commonly encountered infections in outpatients. The distinction between upper UTI and lower UTI is significant as it has therapeutic and prognostic importance. The accurate diagnosis of UTI, especially its anatomical location, is essential for administering anti-microbial therapy successfully. Plasma levels of C-reactive protein (CRP) increase 1,000-fold during bacterial infections. Elevated CRP levels are often seen in acute pyelonephritis and rarely in cystitis. Therefore, this study aimed to evaluate the diagnostic role of serum CRP in upper and lower UTI in adult patients along with correlating its role in UTI patients.

Materials and methods: The study included 81 patients who were >18 years old, diagnosed with UTI by microbiological culture and ultrasonography of the pelvis. Demographic data along with findings of the systemic examination, complete blood count, random blood sugar, urine analysis, and CRP levels of all patients were recorded. Data were analyzed using R Studio.3.5.3 software, and a p-value ≤ 0.05 was considered significant.

Results: The prevalence of UTI was higher among females (60.49%). About 53.09% of patients exhibited upper and 46.91% exhibited lower UTI. The prevalence of *Escherichia coli* was higher among cases of both upper (88.37%) and lower (65.79%) UTI. The most common final diagnosis in upper UTI patients was acute pyelonephritis (90.7%) and that in lower UTI was cystitis (65.79%). The difference in the CRP levels between the upper and lower UTI patients was significant (p=0.02). Thirty-four patients had CRP levels >100 mg/L, all exhibiting upper UTI.

Conclusion: About 53.09% of patients exhibited upper UTI and were diagnosed with acute pyelonephritis. A significant increase in the CRP levels in upper UTI can help determine the anatomical location and can help in targeting effective management of the infection by anti-microbial therapy.

## Introduction

Urinary tract infections (UTIs) are one of the most commonly encountered infections in the patients attending the clinics. It often affects women, with a lifetime incidence of 50%-60%, as the organisms colonizing the vagina and perianal region easily ascend into the urinary system, affecting both the upper and lower regions [1-4]. The predominant pathogen causing UTI is *Escherichia coli*, followed by *Staphylococcus saprophyticus*, *Enterococcus faecalis*, and occasionally *Klebsiella pneumoniae* and *Proteus mirabilis* [5]. Clinical manifestations can range from a simple fever with chills to severe sepsis with septic shock, including urethritis, cystitis, pyelonephritis, and bacteriuria, with accompanying symptoms like frequent urination, dysuria, cloudy and foul-smelling urine, suprapubic pain and/or lower abdominal inconvenience and flank pain, causing prolonged morbidity and occasional mortality, depending upon the underlying host and pathogenic risk factors [6,7].

The accurate diagnosis of UTI, especially its anatomical location, is essential as it affects the type, duration, and route of administration of anti-microbial therapy [8]. The various recommended diagnostic approaches for UTI such as the physical examination, clinical history and laboratory data (blood and urine analysis) are often inconclusive in localizing the site of UTI [9]. Evidence of serum antibodies against bacteria, determination of antibody-coated bacteria in urine, ureteral catheterization, bladder wash-out test, and gallium scans contribute to localizing UTI [9]. Nevertheless, these techniques are not preferred on a routine basis because they are time-consuming, expensive, and are partly invasive methods [9-11].

One of the indirect and non-invasive methods of localizing UTI to the upper or lower tract is evaluating the levels of C-reactive protein (CRP) in blood [9]. It is a homopentameric acute-phase inflammatory protein, that exhibits elevated expressions during inflammatory conditions such as cardiovascular diseases, rheumatoid arthritis, and infections [12,13]. In healthy individuals, CRP levels are 0.3-0.6 mg/dL or 3-6 mg/L [14,15]. During inflammation, the plasma levels of CRP deviate by at least 25%, causing an increase in the circulating levels of CRP by up to 1,000-fold due to bacterial infections [16,17]. The plasma half-life of CRP is 19 hours and is constant under all conditions of health and disease so the sole determinant of circulating CRP concentration is the synthesis rate, which thus reflects the intensity of pathological processes stimulating CRP production [15]. Laser nephelometry and immunoassays are the commonly used methods to quantify CRP levels that are cheap, accurate, and fast [15]. Since CRP plays a vital role in UTI, we aimed our study to evaluate the role of serum CRP and its correlation with upper and lower UTI in adult patients.

## Materials and methods

The prospective observational study was conducted at a tertiary health care center in Bengaluru after obtaining approval from the Institutional Ethics Committee from MS Ramaiah Medical College and Hospital with the letter no. STD-1EC/023/2015 and written informed consent from the patients. The study included 81 patients who were >18 years old and diagnosed with UTI by microbiological culture and ultrasonography (USG). The study excluded patients who were admitted with trauma, pregnant women, and those with elevated CRP levels for reasons other than genitourinary etiologies.

The sample size for this study was calculated based on the proportionality formula from a previously conducted study [18]. The formula used was, with an expected prevalence of 0.5, precision of 11%, a margin of error of 5%, and a sample size of 80 was calculated for this study. However, we recruited 81 patients for our study.

Demographic details such as age, gender, and body mass index (BMI), along with detailed history were taken. Systemic examination and routine investigations were considered complete blood count, random blood sugar, urine routine analysis (urine routine and urine C/S), CRP, renal function test, blood culture, and pelvic ultrasound (USG pelvis) of all patients. Moreover, the present study also noted the prevalence of CRP based on ranges 3-50, 50-100, and >100 mg/L to establish the severity of UTI in patients.

Statistical analysis

Statistical analysis was performed using R Studio.3.5.3 software. The demographic data were expressed as numbers (%). The association of the demographic data and clinical findings with respect to the UTI was calculated by chi-square test and was expressed as Mean ± SD. The level of significance was set at P ≤ 0.05.

## Results

All patients successfully completed the study. Most of the patients were in the age group between 50 and 59 years (26, 32.1%). A total of 49 patients (60.49%) were females and 32 patients (39.51%) were males (p=0.001). The mean age of patients diagnosed with upper UTI was 52.05±10.36 and lower UTI was 46.23±12.75 years. On comparing BMI, 36 patients (44.44%) were found to be in the obese category. The mean BMI of patients diagnosed with upper UTI was 28.41±5.61 and lower UTI was 29.63±5.71 kg/m^2^. However, an association of BMI with UTI was noted to be insignificant (p=0.124). A total of 43 patients (53.09%) comprising 19 females (44.19%) and 24 males (55.81%) had upper UTI whereas 38 patients (46.91%) consisting of 30 females (61.22%) and eight males (25%) were suffering from lower UTI. In patients with upper UTI, 39 patients (90.7%) had a diagnosis of acute pyelonephritis while cystitis was a common diagnostic presentation in 25 patients (65.79%) with lower UTI (Table 1).

**Table 1:** Association of the demographic data and clinical characteristics of the patients with urinary tract infection *Significant; BMI: Body Mass Index; UTI: Urinary Tract Infection; BPH: Benign Prostatic Hyperplasia; USG: Ultrasound findings

Parameters		Number of patients (%)			P-value
		Upper UTI (n=43)	Lower UTI (n=38)	Total (n=81)	
Age (years)	20-29	1 (2.33)	5 (13.16)	6 (7.41)	0.239
	30-39	6 (13.95)	8 (21.05)	14 (17.28)	
	40-49	12 (27.91)	11 (28.95)	23 (28.4)	
	50-59	17 (39.53)	9 (23.68)	26 (32.1)	
	60-69	7 (16.28)	5 (13.16)	12 (14.81)	
Gender	Female	19 (44.19)	30 (78.95)	49 (60.49)	0.001*
	Male	24 (55.81)	8 (21.05)	32 (39.51)	
BMI (kg/m^2^)	Under weight	0	2 (5.26)	2 (2.47)	0.124
	Normal weight	14 (32.56)	6 (15.79)	20 (24.69)	
	Overweight	13 (30.23)	10 (26.32)	23 (28.4)	
	Obese	16 (37.21)	20 (52.63)	36 (44.44)	
Diabetes Mellitus	No	33 (76.74)	20 (52.63)	53 (65.43)	0.023*
	Yes	10 (23.26)	18 (47.37)	28 (34.57)	
Hypertension	No	39 (90.7)	35 (92.11)	74 (91.36)	0.822
	Yes	4 (9.3)	3 (7.89)	7 (8.64)	
Other risk factor	Nil	42 (97.67)	37 (97.37)	79 (97.53)	0.365
	BPH	0	1 (2.63)	1 (1.23)	
	Nephrolithiasis	1 (2.33)	0	1 (1.23)	
Bacteria growth distribution	Citrobacter spp.	0 (0)	2 (5.26)	2 (2.47)	0.132
	Escherichia coli	38 (88.37)	25 (65.79)	63 (77.78)	
	Enterococcus spp.	1 (2.33)	2 (5.26)	3 (3.7)	
	Klebsiella spp.	4 (9.3)	8 (21.05)	12 (14.81)	
	Proteus spp.	0	1 (2.63)	1 (1.23)	
USG pelvis	Normal	0	20 (52.63)	20 (24.69)	0.02*
	Abnormal echogenicity of renal parenchyma	41 (95.35)	0	41 (50.62)	
	Bladder outlet obstruction	0	3 (7.89)	3 (3.7)	
	Bladder wall thickening	0	15 (39.47)	15 (18.52)	
	Emphysematous pyelonephritis	2 (4.65)	0	2 (2.47)	
Diagnosis	Acute pyelonephritis	39 (90.7)	0	39 (48.15)	0.03*
	Cystitis	0	25 (65.79)	25 (30.86)	
	Perinephric abscess	4 (9.3)	0	4 (4.94)	
	Prostatitis	0	3 (7.89)	3 (3.7)	
	Urethritis	0	10 (26.32)	10 (12.35)	

The mean values of the clinical parameters assessed, such as C-reactive protein (CRP), hemoglobin (Hb), creatinine levels, along with the total leucocyte count (TLC) and pus cell count in patients based on the site of UTI is tabulated in Table 2. However, only CRP (p=0.02) and TLC (p=0.03) were significant based on the site of UTI in patients (Table 2).

**Table 2:** Mean values of the clinical parameters in upper and lower UTI patients UTI: Urinary Tract Infection; CRP: C-reactive protein; Hb: Haemoglobin; TLC: Total Leucocyte Count; hpf: high power field

Parameters	Normal Reference Values	Mean±SD		P-value
		Upper UTI	Lower UTI	
CRP (mg/L)	Less than 0.3 mg/L	113.48±40.69	12.84±20.46	0.02*
Hb (g/dL)	12-16 g/dL	13.53±1.97	13.18±2.12	0.563
Creatinine (mg/dL)	0.5-1.2 mg/dL	1.27±0.39	1.32±0.38	0.762
TLC	4,000-10,000 mm^3^	13.81±3.14	8.01±2.44	0.03*
Pus cells	0-5/hpf	26.51±19.35	25.92±17.54	0.692

The prevalence of CRP was tabulated based on ranges 3-50, 50-100, and >100 mg/L to establish the severity of UTI in patients. The CRP levels were between 3 and 50 mg/L in 39 (48.15%), and >100 mg/L in 34 (41.97%) patients. A greater number of patients with CRP >100 mg/L were of the age group 50-59 years (Table 3). All patients with CRP >100 mg/L were diagnosed with upper UTI. The association between the CRP levels with age (p=0.03), gender (p=0.013), site of UTI (p=0.01), ultrasound findings (p=0.01), and the final diagnosis (p=0.02) of the patients in this study was significant (Table 3). The majority of the upper UTI patients diagnosed with acute pyelonephritis (30, 88.24%) had CRP >100 mg/L and lower UTI patients diagnosed with cystitis (22, 62.86%) had CRP levels between 3 and 50 mg/L (Table 3).

**Table 3:** Association of the demographic data and clinical characteristics of the patients with the prevalence of C-reactive protein *Significant; BMI: Body Mass Index; CRP: C-reactive protein; UTI: Urinary Tract Infection; BPH: Benign Prostatic Hyperplasia; USG: Ultrasound findings

Parameters		Number of Patients (%)			P-value
		CRP: 3-50 (mg/L) (N=39)	CRP: 50-100 (mg/L) (N=8)	CRP: >100 (mg/L) (N=34)	
Age (years)	20-29	4 (10.26)	2 (25)	0	0.03*
	30-39	11 (28.21)	0	3 (8.82)	
	40-49	9 (23.08)	4 (50)	10 (29.41)	
	50-59	10 (25.64)	2 (25)	14 (41.18)	
	60-69	5 (12.82)	0	7 (20.59)	
Gender	Female	30 (76.92)	3 (37.5)	16 (47.06)	0.013*
	Male	9 (23.08)	5 (62.5)	18 (52.94)	
BMI (kg/m^2^)	Underweight	2 (5.13)	0	0	0.53
	Normal	7 (17.95)	2 (25)	11 (32.35)	
	Overweight	10 (25.64)	2 (25)	11 (32.35)	
	Obese	20 (51.28)	4 (50)	12 (35.29)	
UTI	Lower UTI	35 (89.74)	3 (37.5)	0	0.01*
	Upper UTI	4 (10.26)	5 (62.5)	34 (100)	
Diabetes Mellitus	No	21 (53.85)	6 (75)	26 (76.47)	0.107
	Yes	18 (46.15)	2 (25)	8 (23.53)	
Hypertension	No	36 (92.31)	7 (87.5)	31 (91.18)	0.906
	Yes	3 (7.69)	1 (12.5)	3 (8.82)	
Other risk factor	Nil	38 (97.44)	8 (100)	33 (97.06)	0.651
	BPH	1 (2.56)	0	0	
	Nephrolithiasis	0	0	1 (2.94)	
Bacteria growth distribution	Citrobacter spp.	2 (5.13)	0	0	0.32
	Escherichia coli	25 (64.1)	7 (87.5)	31 (91.18)	
	Enterococcus spp.	2 (5.13)	0	1 (2.94)	
	Klebsiella spp.	9 (23.08)	1 (12.5)	2 (5.88)	
	Proteus spp.	1 (2.56)	0	0	
USG pelvis	Normal	20 (51.28)	0	0	0.01*
	Abnormal echogenicity of renal parenchyma	4 (10.26)	5 (62.5)	32 (94.12)	
	Bladder outlet obstruction	2 (5.13)	1 (12.5)	0	
	Bladder wall thickening	13 (33.33)	2 (25)	0	
	Emphysematous pyelonephritis	0	0	2 (5.88)	
Diagnosis	Acute pyelonephritis	4 (10.26)	5 (62.5)	30 (88.24)	0.02*
	Cystitis	22 (56.41)	3 (37.5)	0	
	Perinephric abscess	0	0	4 (11.76)	
	Prostatitis	3 (7.69)	0	0	
	Urethritis	10 (25.64)	0	0	

## Discussion

It is vital to differentiate between upper and lower UTI, mainly because of the prognostic and therapeutic consequences. One of the most important indirect, practical, and non-invasive tests to localize UTI into upper or lower tract is to estimate serum CRP levels [19,20].

In the present study, the diagnostic role of CRP among upper and lower UTI patients was evaluated. A greater number of patients in the study belonged to the age group of 50-59 years. Similar observations were reported in a study conducted by Karishetti et al. [21]. The prevalence of UTI was noted to be higher in females (60.49%). Comparable observations have been reported in earlier studies by Mamatha et al. [11] and Agrawal et al. [18], where the prevalence was 66.15% and 65.52%, respectively. The reason for this higher prevalence is because women have a shorter urethra due to which the perineal and fecal flora have a shorter distance to travel [5]. Potential urinary pathogens from the bowel or vagina (during sexual activity), colonize the periurethral mucosa, ascending to the bladder through the urethra and in some cases, to the kidney through the ureter [10]. Therefore, urine factors, urine osmolality, sexual factors, intraoral factors, vaginal pH, and secretor state are causes of the higher prevalence of UTI in women [22,23].

A greater number of patients in the study were diagnosed with upper UTI (53.09%). On the contrary, studies conducted by Mamatha et al. [11] and Bharath et al. [20] reported that 60% and 63.33% of the patients, respectively, were diagnosed with lower UTI. With respect to the demographic data of study participants, a significant association was observed only between gender and site of UTI in patients. Similar results were noted in a study conducted by Patra et al. [24]. Mean CRP levels in upper UTI were 113.48±40.69 mg/L whereas for lower UTI it was 12.84±20.46 mg/L, which was similar to other studies.

A higher number of patients exhibited flank pain, fever, dysuria, and urgency which are some common symptoms that UTI patients experience [21,25]. A greater number of patients were non-diabetic in this study, with only 10 out of 43 upper UTI patients being diabetic. A significant association was observed between the Diabetes Mellitus site of UTI in patients (p=0.023). A similar association (p=0.016) was reported by Odoki et al. [26].

The prevalence of *E. coli* was higher in both upper (88.37%) and lower (65.79%) UTI. A similar prevalence of *E. coli* has been reported in previously conducted studies [18,26-28]. *E. coli* is a pathogen that is reported to be predominant in acute, community-acquired, uncomplicated UTIs in all age groups. This is due to the inherent virulence factors of *E. coli* and its ability to colonize the urinary tract along with its ability to associate with other microbes [29,30]. The ultrasound findings from this study revealed a greater number of patients with upper UTI (95.35%) exhibiting abnormal echogenicity of the renal parenchyma and those with lower UTI (39.47%) exhibiting bladder wall thickening. The final diagnosis in the majority of the patients with upper UTI (90.7%) was acute pyelonephritis and for those with lower UTI (65.79%) was cystitis. The association between the final diagnosis and the site of UTI in patients was significant (p=0.02). Acute pyelonephritis and cystitis are most diagnosed for upper and lower UTI, respectively, because of the bacterial colonization in the urinary tract and may occur as complicated and uncomplicated infections, depending on patient risk factors. Although cystitis can progress to acute pyelonephritis, this occurrence is rare [31,32].

The difference in CRP levels between patients with upper and lower UTI was significant (p=0.02), and comparable observations have been reported in studies conducted earlier [11,18,20]. In previously conducted studies, the usefulness of sequential determination of CRP in acute childhood pyelonephritis was evaluated, that later helped clinicians distinguish between CRP levels in upper and lower UTI [33,34]. The difference in other clinical parameters such as hemoglobin, creatinine levels, and pus cell count between upper and lower UTI patients was insignificant (p>0.05). However, TLC (p=0.03) differed significantly between patients with upper and lower UTI, as these cells are involved in triggering systemic inflammatory response [35].

To establish the severity of the inflammatory protein, the prevalence of CRP levels in UTI patients was delineated as ranging from 3 to 50 (n=39), 50-100 (n=8), and >100 (n=34) mg/L, and the varying study parameters were described based on these CRP levels. The majority of the upper UTI patients (34/43) exhibited CRP levels >100 mg/L, whereas the majority of the lower UTI patients (35/38) exhibited lower CRP levels ranging from 3-50 mg/L. Earlier studies have reported CRP levels ranging from 126.6 to 127.33 mg/L in upper UTI and from 4.7 to 14.5 mg/L in lower UTI [11,18,20]. Association of CRP levels with age (p=0.03) and gender (p=0.013) of UTI patients was also significant in the present study. A study conducted by Al-Khikani et al. [7] reported a significant association between CRP levels and gender (p=0.000) but not age (p=1.38) of UTI patients. A majority of 14 (41.18%) patients belonging to the age group 50-59 years had CRP >100 mg/L in the present study, while CRP levels of >100 mg/L were higher among males (n=18).

Regarding the various clinical characteristics considered for the study, 32 patients with abnormal echogenicity of renal parenchyma as per USG pelvis had CRP levels >100 mg/L, and 13 (33.33%) patients with bladder wall thickening exhibited CRP levels between 3-50 mg/L. With these findings, patients (30, 88.24%) diagnosed with acute pyelonephritis had CRP levels >100 mg/L, while patients (22, 56.41%) diagnosed with cystitis had CRP levels ranging between 3-50 mg/L. Therefore, the association between CRP levels with USG pelvis (p=0.01) and final diagnosis, i.e., upper, or lower UTI (p=0.02) was significant. However, the association between CRP levels and the final diagnosis within the groups of patients with upper UTI (p=0.558) and lower UTI (p=0.429) was insignificant.

Limitations

Firstly, further research with a larger sample size is recommended as an increased number of cases can more precisely predict the outcome of the study. Moreover, larger sample size also helps in correlating the CRP levels with varying pathological conditions.

## Conclusions

Most patients in this study exhibited upper UTI and were diagnosed with acute pyelonephritis. Elevated serum CRP levels (>100 mg/L) were significant in upper UTI patients, whereas no lower UTI patient had such elevated CRP levels. CRP hence proves to be a good diagnostic tool and can be considered an economically feasible, indirect, and non-invasive method to detect UTIs even in peripheral setups to differentiate upper UTIs from lower UTIs for specific therapy and prevent morbidities.

## References

[1] Medina M, Castillo-Pino E (2019). An introduction to the epidemiology and burden of urinary tract infections. Ther Adv Urol.

[2] Mabeck CE (1972). Treatment of uncomplicated urinary tract infection in non-pregnant women. Postgrad Med J.

[3] Gupta V, Nag D, Garg P (2017). Recurrent urinary tract infections in women: how promising is the use of probiotics?. Indian J Med Microbiol.

[4] Christy VR, Athinarayanan G, Mariselvam R (2019). Epidemiology of urinary tract infection in south India. Biomed Res Clin Pract.

[5] Johnson CC (1991). Definitions, classification, and clinical presentation of urinary tract infections. Med Clin North Am.

[6] Marschall J, Zhang L, Foxman B, Warren DK, Henderson JP (2012). Both host and pathogen factors predispose to Escherichia coli urinary-source bacteremia in hospitalized patients. Clin Infect Dis.

[7] AL-Khikani FH, Auda Ga, Ayit AS (2019). Correlation study between urinary tract bacterial infection and some acute inflammatory responses. Biomed Biotechnol Res J.

[8] Ayazi P, Mahyar A, Hashemi HJ (2009). Comparison of procalcitonin and C-reactive protein tests in children with urinary tract infection. Iran J Pediatr.

[9] Khan F, Malik MA, Afzal K (2004). Renal biometry and serum c-reactive protein levels in the evaluation of urinary tract infections. Indian J Nephrol.

[10] Mushi MF, Alex VG, Seugendo M, Silago V, Mshana SE (2019). C - reactive protein and urinary tract infection due to Gram-negative bacteria in a pediatric population at a tertiary hospital, Mwanza, Tanzania. Afr Health Sci.

[11] Mamatha G, Gandhi MVV, Phanindra M (2020). Role of C-reactive protein levels in differentiating upper urinary tract infection and lower urinary tract infection in adults. J Med Sci Clin Res.

[12] Du Clos TW, Mold C (2004). C-reactive protein: an activator of innate immunity and a modulator of adaptive immunity. Immunol Res.

[13] Sproston NR, Ashworth JJ (2018). Role of C-reactive protein at sites of inflammation and infection. Front Immunol.

[14] Chan T, Gu F (2011). Early diagnosis of sepsis using serum biomarkers. Expert Rev Mol Diagn.

[15] Nehring SM, Goyal A, Bansal P (2020). C Reactive Protein (CRP). https://www.ncbi.nlm.nih.gov/books/NBK441843/.

[16] Gabay C, Kushner I (1999). Acute-phase proteins and other systemic responses to inflammation. N Engl J Med.

[17] Thompson D, Pepys MB, Wood SP (1999). The physiological structure of human C-reactive protein and its complex with phosphocholine. Structure.

[18] Agrawal P, Pandey A, Sompura S (2013). Role of blood C - reactive protein levels in upper urinary tract infection and lower urinary tract infection in adult patients (>16 years). J Assoc Physicians India.

[19] Bagga A (2001). Urinary tract infections: evaluation and treatment. Indian J Pediatr.

[20] Bharath MS, Hiremath RS, Basu A (2017). Role of procalcitonin and C-reactive protein in urinary tract infection diagnosis in adults. Int J Adv Med.

[21] Karishetti MS, Shaik HB (2019). Clinicomicrobial assessment of urinary tract infections in a tertiary care hospital. Indian J Health Sci Biomed Res.

[22] Hooton TM (2012). Clinical practice. Uncomplicated urinary tract infection. N Engl J Med.

[23] Almukhtar SH (2018). Urinary tract infection among women aged (18-40) years old in Kirkuk city, Iraq. Open Nursing J.

[24] Patra EP, Karna S, Meher D (2019). Bacterial causes of community-acquired and nosocomial urinary tract infection in type 2 diabetes: a comparative approach. J Diabetol.

[25] Tan CW, Chlebicki MP (2016). Urinary tract infections in adults. Singapore Med J.

[26] Odoki M, Almustapha Aliero A, Tibyangye J (2019). Prevalence of bacterial urinary tract infections and associated factors among patients attending hospitals in Bushenyi district, Uganda. Int J Microbiol.

[27] Singh B, Katiyar D, Tilak R (2018). Prevalence of urinary tract infection causing microorganism and determination of susceptibility to antibiotic among slum women of district Varanasi, India. Int J Curr Microbiol Appl Sci.

[28] Sewify M, Nair S, Warsame S (2016). Prevalence of urinary tract infection and antimicrobial susceptibility among diabetic patients with controlled and uncontrolled glycemia in Kuwait. J Diabetes Res.

[29] Andabati G, Byamugisha J (2010). Microbial aetiology and sensitivity of asymptomatic bacteriuria among ante-natal mothers in Mulago hospital, Uganda. Afr Health Sci.

[30] Tarchouna M, Ferjani A, Ben-Selma W, Boukadida J (2013). Distribution of uropathogenic virulence genes in Escherichia coli isolated from patients with urinary tract infection. Int J Infect Dis.

[31] Knottnerus BJ, Geerlings SE, Moll van Charante EP, ter Riet G (2013). Women with symptoms of uncomplicated urinary tract infection are often willing to delay antibiotic treatment: a prospective cohort study. BMC Fam Pract.

[32] Kang CI, Kim J, Park DW (2018). Clinical practice guidelines for the antibiotic treatment of community-acquired urinary tract infections. Infect Chemother.

[33] Jodal U, Hanson LA (1976). Sequential determination of C-reactive protein in acute childhood pyelonephritis. Acta Paediatr Scand.

[34] Hellerstein S, Duggan E, Welchert E (1982). Serum C-reactive protein and the site of urinary tract infections. J Pediatr.

[35] Leick M, Azcutia V, Newton G, Luscinskas FW (2014). Leukocyte recruitment in inflammation: basic concepts and new mechanistic insights based on new models and microscopic imaging technologies. Cell Tissue Res.

